# 1-(5-Bromo-2-oxoindolin-3-yl­idene)thio­semicarbazone

**DOI:** 10.1107/S1600536813018564

**Published:** 2013-07-13

**Authors:** Katlen C. T. Bandeira, Leandro Bresolin, Christian Näther, Inke Jess, Adriano B. Oliveira

**Affiliations:** a Escola de Química e Alimentos, Universidade Federal do Rio Grande, Av. Itália km 08, Campus Carreiros, 96203-903 Rio Grande-RS, Brazil; b Institut für Anorganische Chemie, Christian-Albrechts-Universität zu Kiel, Max-Eyth Strasse 2, D-24118 Kiel, Germany; c Departamento de Química, Universidade Federal de Sergipe, Av. Marechal Rondon s/n, Campus, 49100-000 São Cristóvão-SE, Brazil

## Abstract

The title mol­ecule, C_9_H_7_BrN_4_OS, is essentially planar [r.m.s. deviation = 0.066 (2) Å], the maximum deviation from the mean plane through the non-H atoms being 0.190 (3) Å for the terminal amine N atom. In the crystal, mol­ecules are linked through N—H⋯O and N—H⋯S inter­actions, generating infinite chains along the *b*-axis direction. In turn, the chains are stacked along the *a* axis *via* π–π inter­actions [centroid–centroid distance = 3.470 (2) Å] and further connected by N—H⋯Br inter­actions into a three-dimensional network. An intra­molecular N—H⋯O hydrogen bond is also observed.

## Related literature

For the pharmacological properties of isatin-thio­semicarbazone derivatives against cruzain, falcipain-2 and rhodesain, see: Chiyanzu *et al.* (2003 ▶). For the synthesis of 5-bromo­isatin-3-thio­semicarbazone, see: Campaigne & Archer (1952 ▶). For the crystal structure of 1-(5-bromo-2-oxoindolin-3-yl­idene)thio­semicarbazide aceto­nitrile monosolvate, see: Pederzolli *et al.* (2011 ▶).
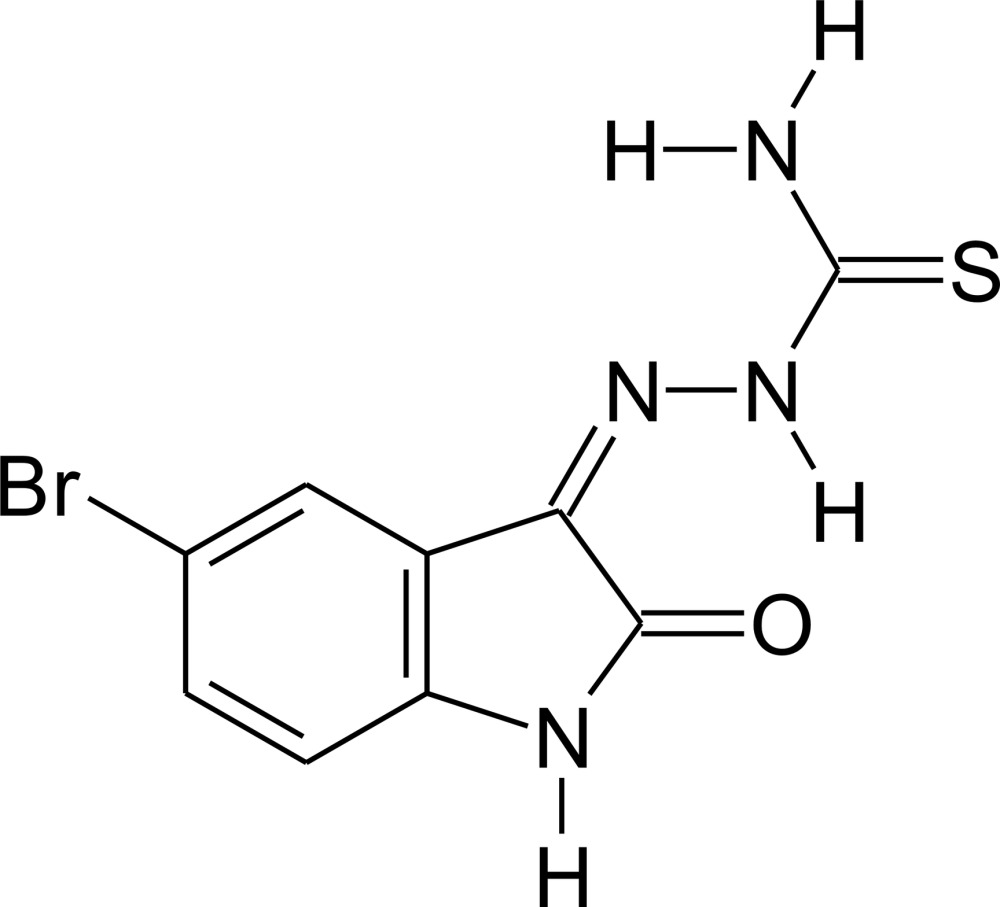


## Experimental

### 

#### Crystal data

C_9_H_7_BrN_4_OS
*M* *_r_* = 299.16
Orthorhombic, 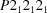

*a* = 4.0185 (2) Å
*b* = 14.6418 (8) Å
*c* = 18.8276 (11) Å
*V* = 1107.78 (10) Å^3^
*Z* = 4
Mo *K*α radiation
μ = 3.88 mm^−1^
*T* = 293 K
0.10 × 0.06 × 0.04 mm

#### Data collection

Stoe IPDS-1 diffractometer
7791 measured reflections
2405 independent reflections
2106 reflections with *I* > 2σ(*I*)
*R*_int_ = 0.051

#### Refinement

*R*[*F* ^2^ > 2σ(*F* ^2^)] = 0.039
*wR*(*F* ^2^) = 0.091
*S* = 1.02
2405 reflections
148 parameters
H-atom parameters constrained
Δρ_max_ = 0.73 e Å^−3^
Δρ_min_ = −0.55 e Å^−3^
Absolute structure: Flack (1983 ▶), 951 Friedel pairs
Absolute structure parameter: −0.015 (13)

### 

Data collection: *X-AREA* (Stoe & Cie, 2008 ▶); cell refinement: *X-AREA*; data reduction: *X-RED32* (Stoe & Cie, 2008 ▶); program(s) used to solve structure: *SHELXS97* (Sheldrick, 2008 ▶); program(s) used to refine structure: *SHELXL97* (Sheldrick, 2008 ▶); molecular graphics: *DIAMOND* (Brandenburg, 2006 ▶); software used to prepare material for publication: *publCIF* (Westrip, 2010 ▶).

## Supplementary Material

Crystal structure: contains datablock(s) I, publication_text. DOI: 10.1107/S1600536813018564/lr2109sup1.cif

Structure factors: contains datablock(s) platon_shelxl. DOI: 10.1107/S1600536813018564/lr2109Isup2.hkl

Additional supplementary materials:  crystallographic information; 3D view; checkCIF report

## Figures and Tables

**Table 1:** Hydrogen-bond geometry (Å, °)

*D*—H⋯*A*	*D*—H	H⋯*A*	*D*⋯*A*	*D*—H⋯*A*
N1—H1⋯S1^i^	0.86	2.82	3.507 (3)	139
N3—H3⋯O1	0.86	2.04	2.726 (4)	135
N4—H2*N*4⋯Br1^ii^	0.83	2.91	3.665 (4)	152
N4—H1*N*4⋯O1^iii^	0.87	1.99	2.851 (4)	167

Symmetry codes: (i) 
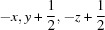
; (ii) 
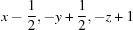
; (iii) 
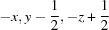
.

**Fig. 1.**
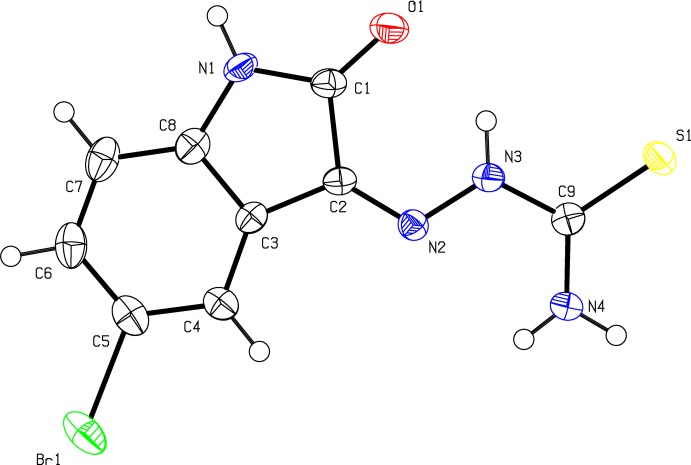
The molecular structure of the title compound with displacement ellipsoids drawn at the 40% probability level.

**Fig. 2.**
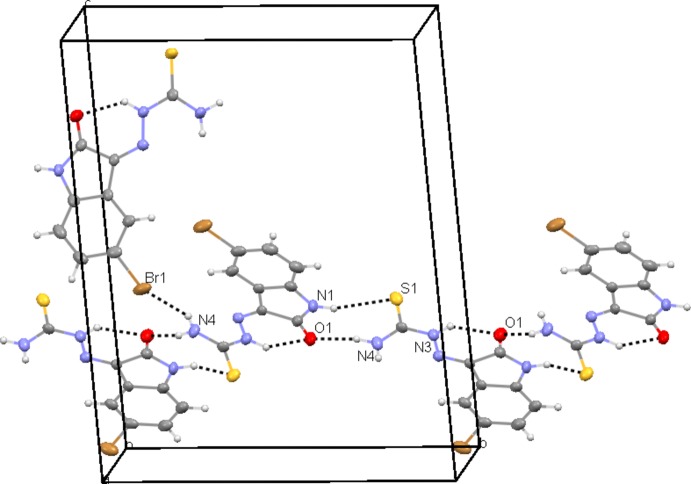
Molecules of the title compound connected via N—H···S, N—H···O and N—H···Br interactions. H-interactions are indicated as dashed lines and the Figure is simplified for clarity.

**Fig. 3.**
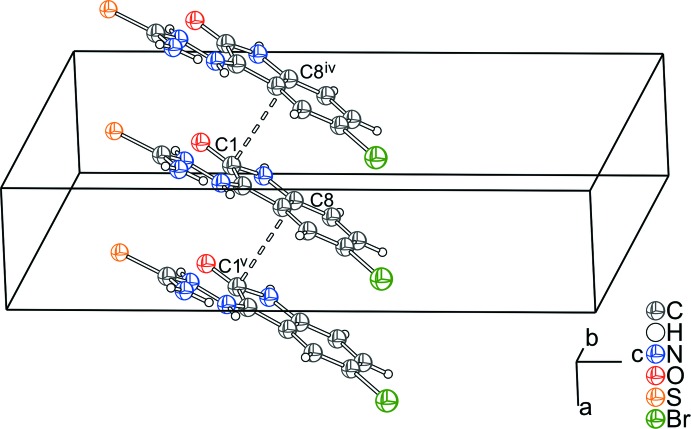
A view of the stacking along the crystallographic a-axis. The π–π-interactions are drawn as dashed lines.Symmetry codes are: (iv) x - 1,y,z; (v) x + 1,y,z.

## supplementary crystallographic information

### Comment

Thiosemicarbazone derivatives have a wide range of biological properties. For example, isatin-based synthetic thiosemicarbazones show pharmacological activity against cruzain, falcipain-2 and rhodesain (Chiyanzu *et al.*, 2003). As part of our study of thiosemicarbazone derivatives, we report herein the crystal structure of 5-bromoisatin-3-thiosemicarbazone (Campaigne & Archer, 1952). In the title compound, in which the molecular structure matches the asymmetric unit, the maximal deviation from the least squares plane through all non-hydrogen atoms amount to 0.1896 (32) Å for N4.The molecule shows an *E* conformation for the atoms about the N2—N3 bond (Fig. 1). The *E* conformation for the thiosemicarbazone fragment is also observed in the crystal structure of the 5-bromoisatin-3-thiosemicarbazone acetonitrile monosolvate (Pederzolli *et al.*, 2011) and is related with the intramolecular N—H···O H-interaction (Table 1). The mean deviations from the least squares planes for the C1—C8/Br1/N1 and C9/N2—N4/S1 fragments amount to 0.0568 (26) Å for O1 and 0.0394 (27) Å for N3, respectively, and the dihedral angle between the two planes is 9.01 (12)°. The molecules are connected *via* centrosymmetric pairs of N—H···S and N—H···O interactions and additionally by N—H···Br interactions (Fig. 2 and Table 1) forming a three-dimensional hydrogen-bonded network, which stabilizes the crystal packing. Additionally, π–π-interactions are observed, with C···C distances = 3.396 (6) Å. The molecules are arranged in layers and are stacked into the crystallographic *a*-axis direction (Fig. 3).

### Experimental

Starting materials were commercially available and were used without further purification. The 5-bromoisatine-3-thiosemicarbazone synthesis was adapted from a procedure reported previously (Campaigne & Archer, 1952). A mixture of of 5-bromoisatin (8,83 mmol) and thiosemicarbazide (8,83 mmol) in ethanol (50 ml) in the presence of a catalytic amount of hydrochloric acid was refluxed for 6 h. After cooling and filtering, the title compound was obtained. Crystals suitable for X-ray diffraction of 5-bromoisatine-3-thiosemicarbazone were obtained unexpectedly from an unsuccessful reaction of SnCl_2_ dihydrate with the title compound in methanol and dichloromethane by the slow evaporation of the solvents. Elemental analysis(%): Calc. 36.01 C, 2.69 H, 18.67 N, 10.68 S; found 35.95 C, 2.25 H, 18.67 N, 10.60 S.

### Refinement

All non-hydrogen atoms were refined anisotropically. All C—H and N—H atoms were located in difference map but were positioned with idealized geometry and refined isotropically with *U*_iso_(H) = 1.2 *U*_eq_(C) using a riding model with C—H = 0.93 Å for aromatic and N—H = 0.86 Å for methyl H atoms. The terminal N—H atoms were located in difference map, their bond lengths were set to 0.86 Å and afterwards they were refined isotropically with *U*_iso_(H) = 1.5 *U*_eq_(N) using a riding model.

### Crystal data

**Table:**

C_9_H_7_BrN_4_OS	*Z* = 4
*M**_r_* = 299.16	*F*(000) = 592
Orthorhombic, *P*2_1_2_1_2_1_	*D*_x_ = 1.794 Mg m^−^^3^
Hall symbol: P 2ac 2ab	Mo *K*α radiation, λ = 0.71073 Å
*a* = 4.0185 (2) Å	µ = 3.88 mm^−^^1^
*b* = 14.6418 (8) Å	*T* = 293 K
*c* = 18.8276 (11) Å	Prism, yellow
*V* = 1107.78 (10) Å^3^	0.10 × 0.06 × 0.04 mm

### Data collection

**Table:**

Stoe IPDS-1 diffractometer	2106 reflections with *I* > 2σ(*I*)
Radiation source: fine-focus sealed tube, Stoe IPDS-1	*R*_int_ = 0.051
Graphite monochromator	θ_max_ = 27.0°, θ_min_ = 2.6°
φ scans	*h* = −4→5
7791 measured reflections	*k* = −18→17
2405 independent reflections	*l* = −24→24

### Refinement

**Table:**

Refinement on *F*^2^	Hydrogen site location: inferred from neighbouring sites
Least-squares matrix: full	H-atom parameters constrained
*R*[*F*^2^ > 2σ(*F*^2^)] = 0.039	*w* = 1/[σ^2^(*F*_o_^2^) + (0.0516*P*)^2^ + 0.9296*P*] where *P* = (*F*_o_^2^ + 2*F*_c_^2^)/3
*wR*(*F*^2^) = 0.091	(Δ/σ)_max_ < 0.001
*S* = 1.02	Δρ_max_ = 0.73 e Å^−^^3^
2405 reflections	Δρ_min_ = −0.55 e Å^−^^3^
148 parameters	Extinction correction: *SHELXL97* (Sheldrick, 2008), Fc^*^=kFc[1+0.001xFc^2^λ^3^/sin(2θ)]^-1/4^
0 restraints	Extinction coefficient: 0.0123 (16)
Primary atom site location: structure-invariant direct methods	Absolute structure: Flack (1983), 951 Friedel pairs
Secondary atom site location: difference Fourier map	Flack parameter: −0.015 (13)

### Special details

**Table:**

Geometry. All esds (except the esd in the dihedral angle between two l.s. planes) are estimated using the full covariance matrix. The cell esds are taken into account individually in the estimation of esds in distances, angles and torsion angles; correlations between esds in cell parameters are only used when they are defined by crystal symmetry. An approximate (isotropic) treatment of cell esds is used for estimating esds involving l.s. planes.
Refinement. Refinement of *F*^2^ against ALL reflections. The weighted *R*-factor *wR* and goodness of fit *S* are based on *F*^2^, conventional *R*-factors *R* are based on *F*, with *F* set to zero for negative *F*^2^. The threshold expression of *F*^2^ > σ(*F*^2^) is used only for calculating *R*-factors(gt) *etc*. and is not relevant to the choice of reflections for refinement. *R*-factors based on *F*^2^ are statistically about twice as large as those based on *F*, and *R*- factors based on ALL data will be even larger.

### Fractional atomic coordinates and isotropic or equivalent isotropic displacement parameters (Å^2^)

**Table:**

	*x*	*y*	*z*	*U*_iso_*/*U*_eq_	
Br1	1.14379 (13)	0.36317 (4)	0.59001 (2)	0.04209 (17)	
S1	−0.1204 (3)	0.31338 (7)	0.15460 (5)	0.0298 (2)	
O1	0.2574 (8)	0.5699 (2)	0.26587 (16)	0.0352 (8)	
N1	0.5769 (10)	0.6107 (2)	0.36442 (17)	0.0271 (8)	
H1	0.5788	0.6690	0.3590	0.033*	
N2	0.3785 (9)	0.3806 (2)	0.32307 (15)	0.0223 (6)	
N3	0.2026 (9)	0.3844 (2)	0.26195 (17)	0.0248 (7)	
H3	0.1764	0.4359	0.2406	0.030*	
N4	0.0979 (10)	0.2311 (2)	0.27236 (17)	0.0285 (7)	
H1N4	0.0154	0.1799	0.2562	0.029 (13)*	
H2N4	0.1793	0.2242	0.3124	0.038 (15)*	
C1	0.4265 (11)	0.5512 (3)	0.31935 (19)	0.0254 (9)	
C2	0.4870 (10)	0.4577 (2)	0.3483 (2)	0.0209 (7)	
C3	0.6801 (9)	0.4700 (2)	0.4126 (2)	0.0213 (7)	
C4	0.8021 (10)	0.4087 (3)	0.4623 (2)	0.0253 (8)	
H4	0.7701	0.3461	0.4574	0.030*	
C5	0.9739 (11)	0.4448 (3)	0.5197 (2)	0.0299 (9)	
C6	1.0274 (11)	0.5383 (3)	0.5283 (2)	0.0335 (10)	
H6	1.1455	0.5597	0.5674	0.040*	
C7	0.9031 (13)	0.5993 (3)	0.4782 (2)	0.0344 (10)	
H7	0.9363	0.6618	0.4832	0.041*	
C8	0.7292 (10)	0.5647 (3)	0.4210 (2)	0.0269 (9)	
C9	0.0663 (9)	0.3069 (3)	0.2340 (2)	0.0227 (8)	

### Atomic displacement parameters (Å^2^)

**Table:**

	*U*^11^	*U*^22^	*U*^33^	*U*^12^	*U*^13^	*U*^23^
Br1	0.0320 (2)	0.0652 (3)	0.0290 (2)	−0.0030 (3)	−0.0044 (2)	0.0148 (2)
S1	0.0302 (5)	0.0322 (5)	0.0270 (4)	−0.0033 (5)	−0.0081 (5)	0.0052 (4)
O1	0.052 (2)	0.0260 (14)	0.0281 (14)	0.0089 (13)	−0.0046 (13)	0.0020 (12)
N1	0.038 (2)	0.0155 (15)	0.0274 (16)	0.0004 (13)	0.0009 (15)	−0.0012 (11)
N2	0.0221 (15)	0.0232 (15)	0.0218 (13)	0.0011 (14)	−0.0005 (14)	−0.0003 (11)
N3	0.030 (2)	0.0213 (16)	0.0228 (14)	0.0008 (13)	−0.0016 (13)	0.0024 (12)
N4	0.035 (2)	0.0228 (16)	0.0272 (16)	−0.0010 (15)	−0.0076 (16)	0.0050 (12)
C1	0.030 (2)	0.0227 (18)	0.0233 (18)	0.0025 (16)	0.0038 (16)	0.0031 (14)
C2	0.0209 (18)	0.0195 (17)	0.0223 (17)	0.0033 (14)	0.0046 (14)	0.0013 (14)
C3	0.0202 (19)	0.0214 (16)	0.0224 (16)	−0.0034 (14)	0.0063 (17)	−0.0001 (14)
C4	0.020 (2)	0.0313 (19)	0.0241 (17)	−0.0021 (15)	0.0031 (15)	0.0055 (15)
C5	0.021 (2)	0.046 (2)	0.0223 (18)	−0.0016 (17)	0.0043 (15)	0.0033 (17)
C6	0.029 (2)	0.045 (3)	0.027 (2)	−0.0087 (19)	0.0034 (17)	−0.0087 (18)
C7	0.036 (3)	0.036 (2)	0.0306 (19)	−0.010 (2)	0.010 (2)	−0.0104 (16)
C8	0.030 (2)	0.0275 (19)	0.0236 (19)	−0.0019 (15)	0.0086 (15)	−0.0031 (15)
C9	0.018 (2)	0.0256 (18)	0.0248 (17)	−0.0018 (14)	0.0051 (14)	0.0014 (15)

### Geometric parameters (Å, º)

**Table:**

Br1—C5	1.910 (4)	N4—H2N4	0.8285
S1—C9	1.675 (4)	C1—C2	1.494 (5)
O1—C1	1.245 (5)	C2—C3	1.449 (6)
N1—C1	1.358 (5)	C3—C4	1.387 (5)
N1—C8	1.400 (5)	C3—C8	1.409 (5)
N1—H1	0.8598	C4—C5	1.386 (6)
N2—C2	1.299 (5)	C4—H4	0.9300
N2—N3	1.352 (4)	C5—C6	1.396 (6)
N3—C9	1.367 (5)	C6—C7	1.392 (7)
N3—H3	0.8600	C6—H6	0.9300
N4—C9	1.330 (5)	C7—C8	1.381 (6)
N4—H1N4	0.8746	C7—H7	0.9300
			
C1—N1—C8	111.2 (3)	C5—C4—C3	117.1 (4)
C1—N1—H1	124.5	C5—C4—H4	121.4
C8—N1—H1	124.3	C3—C4—H4	121.4
C2—N2—N3	116.8 (3)	C4—C5—C6	122.8 (4)
N2—N3—C9	120.2 (3)	C4—C5—Br1	118.7 (3)
N2—N3—H3	119.9	C6—C5—Br1	118.5 (3)
C9—N3—H3	119.9	C7—C6—C5	119.7 (4)
C9—N4—H1N4	119.3	C7—C6—H6	120.2
C9—N4—H2N4	129.3	C5—C6—H6	120.2
H1N4—N4—H2N4	111.2	C8—C7—C6	118.4 (4)
O1—C1—N1	127.3 (4)	C8—C7—H7	120.8
O1—C1—C2	125.9 (4)	C6—C7—H7	120.8
N1—C1—C2	106.7 (3)	C7—C8—N1	129.6 (4)
N2—C2—C3	126.4 (3)	C7—C8—C3	121.3 (4)
N2—C2—C1	127.4 (4)	N1—C8—C3	109.1 (3)
C3—C2—C1	106.1 (3)	N4—C9—N3	116.4 (3)
C4—C3—C8	120.7 (4)	N4—C9—S1	125.1 (3)
C4—C3—C2	132.3 (3)	N3—C9—S1	118.4 (3)
C8—C3—C2	106.9 (3)		
			
C2—N2—N3—C9	−176.8 (4)	C3—C4—C5—C6	−0.3 (6)
C8—N1—C1—O1	176.5 (4)	C3—C4—C5—Br1	179.5 (3)
C8—N1—C1—C2	−0.5 (5)	C4—C5—C6—C7	0.5 (7)
N3—N2—C2—C3	−179.9 (4)	Br1—C5—C6—C7	−179.3 (3)
N3—N2—C2—C1	3.1 (6)	C5—C6—C7—C8	0.0 (7)
O1—C1—C2—N2	0.6 (7)	C6—C7—C8—N1	179.1 (4)
N1—C1—C2—N2	177.7 (4)	C6—C7—C8—C3	−0.6 (6)
O1—C1—C2—C3	−176.9 (4)	C1—N1—C8—C7	−179.1 (4)
N1—C1—C2—C3	0.2 (4)	C1—N1—C8—C3	0.6 (5)
N2—C2—C3—C4	0.9 (7)	C4—C3—C8—C7	0.8 (6)
C1—C2—C3—C4	178.4 (4)	C2—C3—C8—C7	179.3 (4)
N2—C2—C3—C8	−177.4 (4)	C4—C3—C8—N1	−178.9 (3)
C1—C2—C3—C8	0.1 (4)	C2—C3—C8—N1	−0.4 (4)
C8—C3—C4—C5	−0.3 (6)	N2—N3—C9—N4	4.4 (6)
C2—C3—C4—C5	−178.4 (4)	N2—N3—C9—S1	−174.7 (3)

### Hydrogen-bond geometry (Å, º)

**Table:**

*D*—H···*A*	*D*—H	H···*A*	*D*···*A*	*D*—H···*A*
N1—H1···S1^i^	0.86	2.82	3.507 (3)	139
N3—H3···O1	0.86	2.04	2.726 (4)	135
N4—H2*N*4···Br1^ii^	0.83	2.91	3.665 (4)	152
N4—H1*N*4···O1^iii^	0.87	1.99	2.851 (4)	167

Symmetry codes: (i) −*x*, *y*+1/2, −*z*+1/2; (ii) *x*−1/2, −*y*+1/2, −*z*+1; (iii) −*x*, *y*−1/2, −*z*+1/2.

## References

[bb1] Brandenburg, K. (2006). *DIAMOND* Crystal Impact GbR, Bonn, Germany.

[bb2] Campaigne, E. & Archer, W. L. (1952). *J. Am. Chem. Soc.* **74**, 5801.

[bb3] Chiyanzu, I., Hansell, E., Gut, J., Rosenthal, P. J., McKerrow, J. H. & Chibale, K. (2003). *Bioorg. Med. Chem. Lett.* **13**, 3527–3530. 10.1016/s0960-894x(03)00756-x 14505663

[bb4] Flack, H. D. (1983). *Acta Cryst.* A**39**, 876–881.

[bb5] Pederzolli, F. R. S., Bresolin, L., Carratu, V. S., Locatelli, A. & Oliveira, A. B. de (2011). *Acta Cryst.* E**67**, o1804. 10.1107/S1600536811023786 PMC3152066 21837177

[bb6] Sheldrick, G. M. (2008). *Acta Cryst.* A**64**, 112–122. 10.1107/S0108767307043930 18156677

[bb7] Stoe & Cie (2008). *X-AREA* and *X-RED32* Stoe & Cie, Darmstadt, Germany.

[bb8] Westrip, S. P. (2010). *J. Appl. Cryst.* **43**, 920–925.

